# Fuzheng Huayu Recipe and its active compounds inhibited HBeAg production by promoting TOMM34 gene expression in HBV-infected hepatocytes

**DOI:** 10.3389/fphar.2022.907921

**Published:** 2022-09-26

**Authors:** Lu Xing, Rui Zeng, Kai Huang, Jingbo Xue, Hongliang Liu, Zhimin Zhao, Yuan Peng, Xudong Hu, Chenghai Liu

**Affiliations:** ^1^ Institute of Liver diseases, Shuguang Hospital affiliated to Shanghai University of Traditional Chinese Medicine, Shanghai, China; ^2^ Department of Biology, School of Basic Medical Sciences, Shanghai University of Traditional Chinese Medicine, Shanghai, China; ^3^ Shanghai Key Laboratory of Traditional Chinese Clinical Medicine, Shanghai, China

**Keywords:** Fuzheng Huayu Recipe, HBeAg, TOMM34, active compounds, HBV

## Abstract

**Background and aim:** Fuzheng Huayu Recipe (FZHY) is a Chinese patent medicine (approval No. Z20020074) included in the national medical insurance catalogue, which is mainly used for anti-hepatic fibrosis treatment of hepatitis B virus (HBV) induced liver fibrosis and liver cirrhosis. In clinical practice, we discovered that FZHY might also have a direct anti-HBV effect on inhibiting HBeAg production, but the mechanism underlying was unclear. This study aimed to clarify the molecular mechanism of the inhibition effect of FZHY on HBeAg production.

**Methods:** The decrease degree of serum HBeAg titer in FZHY + entecavir (ETV) group patients were analyzed through clinical data. C57BL/6N-Tg (1.28HBV)/Vst HBV transgenic mice were used for *in vivo* experiments. HepG2. 2.15 cells (wild-type HBV replication cells) were used for *in vitro* experiments.

**Results:** The clinical study results showed that the decrease degree of serum HBeAg titer in FZHY+ETV group was significantly higher than that in ETV group after 48 weeks treatment. *In vivo* experiments results showed that FZHY could significantly reduce the serum HBeAg titer in HBV transgenic mice, and promote HBeAg seroconversion. *In vitro* experiments results showed that FZHY could reduce HBeAg titer dependently, but it did not significantly inhibit the expression of HBsAg and HBV-DNA. Further cell experiments *in vitro* discovered that TOMM34 might be the key target for FZHY to inhibit HBeAg production. The subsequent pharmacological screening experiment of 20 active compounds in FZHY showed that quercetin, baicalin and cordycepin could promote the expression of TOMM34 gene and reduce the production of HBeAg.

**Conclusion:** In conclusion, FZHY and its active compounds quercetin, baicalin and cordycepin could inhibit HBeAg production by promoting the expression of TOMM34 gene in HBV-infected hepatocytes.

## Graphical Abstract

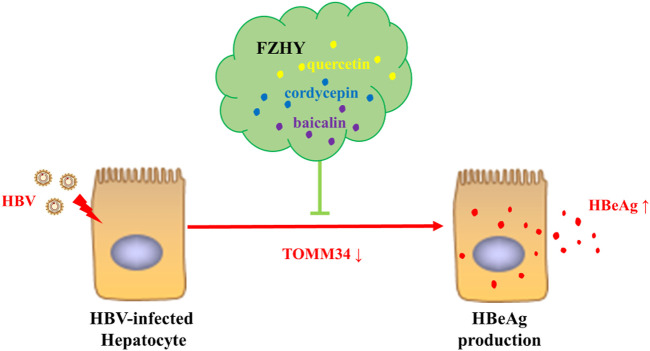

## Introduction

Hepatitis B virus (HBV) has brought a heavy burden on global health. According to WHO estimates, there were 296 million chronic HBV infections worldwide in 2019, with 1.5 million new infections each year. In 2019, hepatitis B caused 820,000 deaths, mainly due to liver cirrhosis and hepatocellular carcinoma (HCC) (WHO, 2021). Around 10% people with HBV infection will be the patients with HBV-related cirrhosis (Tan et al., 2021), at least 50% of liver cancer cases in the world come from chronic HBV infection (Xie, 2017).

Hepatitis B virus e antigen (HBeAg) is a major product of HBV replication in human body, which is encoded by pre-C and C genes. HBeAg is a soluble component of hepatitis B core antigen and a marker of HBV replication and infectivity. As an immunomodulatory factor, HBeAg can regulate the host immune response, inhibit the cytotoxic activity of host T cells, and form immune tolerance to HBV infection (Merkle et al., 2000; Barth et al., 2001; Wilson et al., 2011). HBeAg loss and serum transformation are very important for prognosis. The continuous positive HBeAg in patients with chronic hepatitis B indicates persistent HBV infection, which is a sign of active hepatitis, and the probability of developing cirrhosis is relatively high (Deng et al., 2009). High serum HBeAg levels are associated with the occurrence of liver cirrhosis and HCC (Lin et al., 2007; Liu et al., 2016). Therefore, it is a very important goal of hepatitis B treatment to promote serum HBeAg loss and serum HBeAg conversion.

**FIGURE 1:**
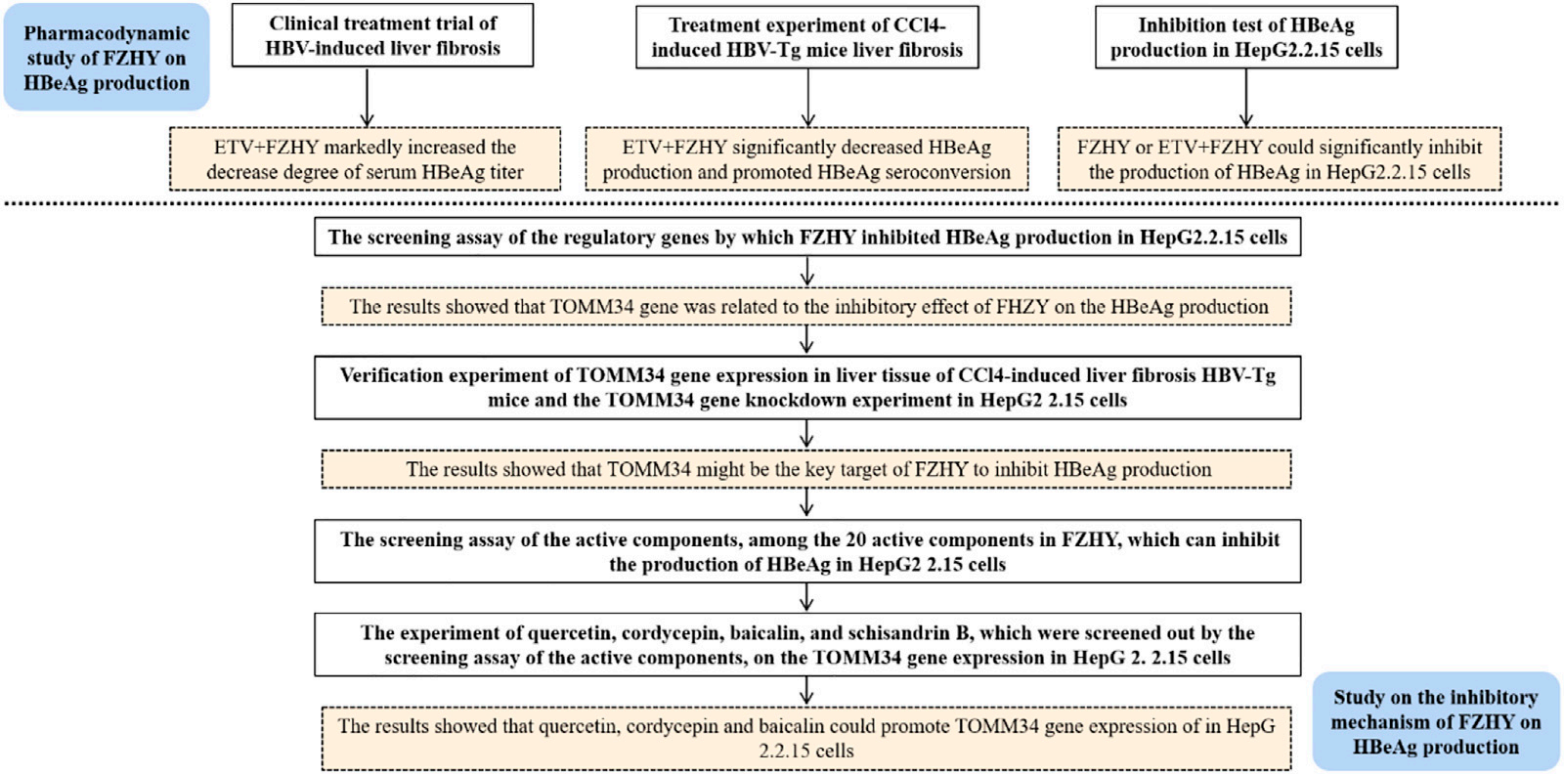
The flowchart of the study.

In recent years, the practice of complementary medicine and alternative medicine including traditional Chinese medicine (TCM) in the treatment of chronic hepatitis B has increased significantly all over the world (Chan et al., 2010). The combination of traditional Chinese medicine and modern medicine has gradually become an important way to treat chronic hepatitis B and its related diseases. In China, clinicians use a variety of traditional Chinese medicine prescriptions to treat chronic hepatitis B liver fibrosis. Fuzheng Huayu Recipe is one of the first-line traditional Chinese medicine prescriptions recommended in the Chinese pharmacopoeia for chronic hepatitis B liver fibrosis. In clinical practice, we discovered that FZHY could inhibit HBeAg production, but the mechanism underlying was unknown. In this study, we aimed to discover the inhibitory mechanism of FZHY on HBeAg production (Figure 1).

## Materials and methods

### Patient acceptance criteria and grouping

The cases were from 5 hospitals including Shuguang Hospital affiliated to Shanghai University of Traditional Chinese Medicine, Zhongshan Hospital Affiliated to Fudan University, Beijing Ditan Hospital Affiliated to Capital Medical University, Beijing Youan Hospital Affiliated to Capital Medical University, and Shijiazhuang Fifth Hospital: clinical study of TCM combined with entecavir (ETV) in the treatment of hepatitis B cirrhosis (2015–445-73–02). All patients were approved by the Ethics Committee. Patient grouping with HBeAg-positive hepatitis B fibrosis which treated for 48 weeks: entecavir + Fuzheng Huayu tablet group (treatment group): 79 cases; entecavir + Fuzheng Huayu placebo group (control group): 77 cases.

### Drug preparation, animal grouping, modeling and administration

Fuzheng Huayu Recipe extract powder (batch No.180,206) was provided by Shanghai Huanghai Pharmaceutical Co., Ltd. Quality control standard: brown powder, bitter and astringent; For each gram of fermented grass fungus powder, adenosine shall not be less than 1000 g/batch, Salvia miltiorrhiza sodium shall not be less than 3000 g/batch, salvianolic acid B shall not be less than 5000 g/batch, water content shall be less than 8.0%, the total number of aerobic bacteria shall not exceed 1000 CFU/g, the total number of molds and yeasts shall not exceed 100 CFU, and *Escherichia coli* shall not be detected. Entecavir (ETV) (cat#21995) was purchased from MCE.

48 male C57BL/6N-Tg (1.28HBV)/Vst HBV transgenic mice (HBV-Tg mice) and 12 male C57BL/6N wild-type mice, weighing (20 ± 5) g, SPF grade, were purchased from and raised in Shanghai Branch of Beijing Weitonglihua Technology Co., Ltd. All mice were given free diet and drinking water.

After 1 week of adaptive feeding of all mice, 48 male HBV-Tg mice were randomly divided into 4 groups (12 mice/group): control group (HBV-Tg control), CCl4 liver fibrosis model group (HBV-Tg CCl4 model), ETV group (HBV-Tg CCl4 model + ETV), and ETV+FZHY group (HBV-Tg CCl4 model+ETV+FZHY). At the same time, 12 male C57BL/6N wild-type mice were used as the wild-type control group (WT control). Mice in HBV-Tg CCl4 model group, HBV-Tg CCl4 model+ETV group, and HBV-Tg CCl4 model+ETV+FZHY group were injected with 10% CCl4 olive oil solution intraperitoneally at the dose of 2 ml/kg mouse body weight, once every other day for 6 weeks. Mice in WT control group and HBV-Tg control group were given the same dose of olive oil at the same time.

After the 6th injection of CCl4, ETV (0.1 mg drug/kg mouse body weight) was administered to HBV-Tg CCl4 model+ETV group and HBV-Tg CCl4 model+ETV+FZHY group mice by gavage, which was dissolved in 0.1% sodium carboxymethyl cellulose solution, once a day for 28 days. FZHY (5.6 g crude drug/kg mouse body weight) was administered to HBV-Tg CCl4 model+ETV+FZHY group by gavage, which was dissolved in double distilled water, at least 4 h apart from ETV administered, once a day for 28 days. WT control group, HBV-Tg control group and HBV-Tg CCl4 model group were given the same amount of solvent by gavage. 48 h after the 18th injection of CCl4, the mice were sacrificed.

### Determination of serum HBsAg, HBeAg, HBeAb and HBV DNA

Serum HBsAg, HBeAg and HBeAb were detected by ELISA according to the operation instructions of the kit. Serum HBV DNA was detected by fluorescence quantitative PCR (probe method), samples were prepared according to the instructions of the kit, and amplified by ABIViiA7 fluorescence quantitative PCR instrument. The diagnostic kits (ELISA) for Hepatitis B Virus Surface Antigen, Hepatitis B Virus e Antigen, Hepatitis B e Antibody and the quantification kit (PCR-Fluorescence Probing) for Hepatitis B Virus DNA were purchased from Shanghai Kehua Bio-Engingeering Co.,Ltd.

### Liver histopathology

The liver tissue of mice was fixed with 10% neutral formaldehyde for 48 h, then dehydrated with gradient alcohol, embedded in paraffin, 4 μM thick section, dewaxing with xylene to water. Finally, HE staining was used to observe the degree of liver inflammation and Sirius red staining was used to observe the collagen deposition in liver tissue.

### Immunohistochemical staining of liver tissue

Mouse liver tissue sections were dewaxed to water, endogenous peroxidase was inactivated by 3% H_2_O_2_, antigen was thermally repaired by citrate buffer microblog boiling method, 5% BSA was blocked, primary antibody was incubated at 4 °C overnight, secondary antibody was incubated at 37°C, DAB color was developed, hematoxylin was lined, alcohol was gradually dehydrated, xylene was transparent, neutral gum was sealed, and observed and photographed under the microscope, Image-Pro Plus software was used for semi quantitative analysis.

### Cell culture and cytotoxicity assay

Caffeic acid (cat#100317), entomolic acid (Lot#WH0834), ergosterol (cat#140627), adenosine (Lot#WH0506), protopanaxadiol (cat#110729), baicalin (cat#110830), rutin (cat#140108), ginsenoside F2 (cat#131229), uridine (cat#130311), tanshinone IIA (cat#200506), salvianolic acid B (cat#200316), cryptotanshinone (cat#140119), sodium tanshinol (Lot#WH0060), protopanaxatriol (cat#150429), quercetin (cat#140413), amygdalin (cat#191227), cordycepin (cat#141124), ginsenoside Rb1 (cat#141128), ginsenoside RB3 (cat#121216), and ginsenoside Rg3 (cat#121229) were purchased from Shanghai Ronghe pharmaceutical. schisandrin B (cat#15110531) was purchased from TAUTO BIOTECH.

Human liver cancer HepG2 2.15 cell line was purchased from BeNa Culture Collection (BNCC). HepG2 2.15 cells were cultured in DMEM medium containing 10% FBS +0.4% G418. Culture conditions: 37°C, 5% CO_2_ and 95% humidity.

1×10^5^/well HepG2.2.15 cells were seeded into 96 well plates, FZHY (6.25, 12.5, 25, 50, 100 μg/ml), ETV (3.125, 6.25, 12.5, 25, 50 μg/ml) or 20 monomers (1, 10, 100 μM caffeic acid, cordycepin acid, ergosterol, adenosine, protopanaxadiol, baicalin, rutin, ginsenoside F2, uridine, tanshinone IIA, salvianolic acid B, cryptotanshinone, Danshensu Sodium, protopanaxatriol, quercetin, amygdalin, cordycepin, schisandrin B, ginsenoside Rb1, ginsenoside Rb3 and ginsenoside Rg3) were administered, 6 duplicate wells were set for each drug. After incubation for 24 h, the supernatant was discarded, 100 μl 10% CCK8 was added to each well and continue to culture for 2 h. Then, the absorbance was measured at the wavelength of 450/630 nm and the cell survival rate was calculated, which was used to evaluate the cytotoxicity of FZHY and 20 monomers. The Cell Counting Kit (CCK8) was purchased from Thermo Fisher Scientific Inc.

### siTOMM34 interference experiment

1.25 μl of 20 μM TOMM34 siRNA storage solution was diluted with 30 μl riboFECT™ CP Buffer and gently mixed. Then, 3 μl riboFECT™ CP Reagent was added, gently blown and mixed, incubated for 15 min at room temperature to prepare transfection complex. Next, transfection complex was added to appropriate amount of non-double antibody complete DMEM medium and gently mixed. 5×10^4^/well HepG2.2.15 cells were seeded into 48-well plate and TOMM34 siRNA was transfected into 48-well plates at the final transfection concentration of 50 nM. Control group, FZHY group, NC group, positive group, siTOMM34 group, ETV group and FZHY+ETV group were set. The 48-well plate was cultured in 5% CO_2_, 37°C for 96 h. Finally, the gene expression of TOMM34 was detected by qPCR, and the expression of HBeAg was detected by ELISA. TOMM34 siRNA and transfection Kit were purchased from Guangzhou RiboBio Co., Ltd.

### qRT-PCR

Cells and liver tissues were collected and lysed by Trizol, total RNA was extract, reverse transcription and amplification kit was used to synthesize and amplify cDNA. 2^−ΔΔCT^ method was used for relative quantitative analysis of gene expression with β-actin internal reference gene. All primers were synthesized by Shanghai Sangong Bioengineering Co. Ltd. The primer sequences were shown in Table 1.

**TABLE 1:** Gene primer sequence.

Gene	Sequence
Human/mouse-βactin-F	TGA​CGA​GGC​CCA​GAG​CAA​GA
Human/mouse-βactin-R	ATG​GGC​ACA​GTG​TGG​GTG​AC
Human-C11orf9-F	GAC​CCC​AAC​TAC​CAG​TCC​ATC
Human-C11orf9-R	TCG​GGC​GTC​TTG​ACG​TAC​T
Human-FJX1-F	CCG​GCT​CGT​AAG​CAA​CCT​C
Human-FJX1-R	AGC​GGC​TCG​TTA​TAC​TTG​TCC
Human-ADCY5-F	TCT​CCT​GCA​CCA​ACA​TCG​TG
Human-ADCY5-R	CAT​GGC​AAC​ATG​ACG​GGG​A
Human-SLA39A11-F	CAG​CTC​TCG​TGT​TCG​TAT​TCT​C
Human-SLA39A11-R	TCA​GCC​AAG​TAG​ACA​AAA​GCC
Human-SETMAR-F	GAA​GCG​GCA​AAG​ACG​ACA​C
Human-SETMAR-R	GAG​TGG​GAT​CAA​TGT​CTG​CTC
Human-TNFAIP3-F	TCC​TCA​GGC​TTT​GTA​TTT​GAG​C
Human-TNFAIP3-R	TGT​GTA​TCG​GTG​CAT​GGT​TTT​A
Human-SRA1-F	CTG​AGG​TCA​GTC​AGT​GGA​TGG
Human-SRA1-R	AGC​CTG​GTA​TGG​TAT​GGT​TCT
Human-HLA-E-F	TTC​CGA​GTG​AAT​CTG​CGG​AC
Human-HLA-E-R	GTC​GTA​GGC​GAA​CTG​TTC​ATA​C
Human-HIGD1A-F	AAG​AGG​CAC​CAT​TCG​TAC​CC
Human-HIGD1A-R	ACC​AAC​AGT​CAT​TGC​TCC​TAC​A
Human-FAM176A-F	AGC​AAC​ATC​CTA​GCG​GCC​TA
Human-FAM176A-R	TGT​GTG​GCA​AGA​GAT​CCT​TAT​CA
Human-TOMM34-F	TGC​ATC​AAA​GAT​TGC​ACT​TCA​GC
Human-TOMM34-R	GCA​GCA​CAG​TCT​TAT​AGT​CAA​CA
Mouse-TOMM34-F	CCT​GGA​AGG​CAT​CAA​CAG​AAT
Mouse-TOMM34-R	GGC​ACT​CTG​CTC​TTC​GTA​GC

### Western blot

Mice liver tissues, HepG2.2.15 cells were collected and lysed with RIPA containing 1% PMSF and 0.01% phosphatase inhibitor for 30min, centrifuged at 4°C and 12,000g for 15 min, and the supernatant was collected. Protein denaturation, electrophoresis, membrane transfer and blocking were performed after protein quantified by BCA method. 1:250 diluted TOMM34 antibody was added and incubated overnight. The fluorescent secondary antibody was incubated in the dark for 60 min at room temperature, and the Odyssey infrared imaging system was used for scanning and reading the target band. The relative quantitative value of TOMM34 protein expression was measured with GAPDH internal reference protein. The Rabbit anti-TOMM34 Polyclonal Antibody was purchased from Absin Bioscience Inc.

### Statistical analysis

The counting data was expressed by frequency. The measurement data with normal distribution were expressed by mean ± standard deviation (x ± s), and those with non-normal distribution were expressed by P50 (P25, P75) quartile. One-way ANOVA was used for pairwise comparison between groups. *P*< 0.05 means the difference is statistically significant.

## Results

### The serum HBeAg titer in ETV+FZHY group decreased significantly than that in ETV group after 48 weeks treatment

156 chronic hepatitis B patients with HBeAg positive were divided into 2 groups according to the treatment method. The baseline data of demographic data, biochemical virology and fibrosis grades were balanced before treatment. By analyzing and screening the clinical cohort data of patients with HBeAg (S/CO) > 1 in the experimental group (FZHY+ETV, *n* = 79) and the control group (placebo+ETV, n = 77), the result showed that the decrease degree of serum HBeAg titer in FZHY+ETV group was significantly higher than that in ETV group after 48 weeks treatment (*p* < 0.05) (Table 2). This result indicated that FZHY could decrease the HBeAg production in chronic hepatitis B patients with HBeAg positive.

**TABLE 2:** The therapeutic outcomes of the two groups after 48 weeks treatment.

	FZHY + ETV n = 79	ETV n = 77	*p* Value
Age, year (mean±SD)	42.20±8.45	42.43±8.41	0.880
Male, n (%)	64 (81.01)	60 (77.92)	0.633
HBeAg, COI (median, IQR) 0–48 weeks	0.18 (-0.02–18.10)	0.01 (-0.04–1.28)	0.039
HBV DNA, Log10 IU/ml (median, IQR) 0–48 weeks	3.97 (3.18–5.67)	3.64 (2.21–5.45)	0.070
ALT, U/L (median, IQR) 0–48 weeks	15.00 (4.00–32.70)	12.00 (1.00–36.25)	0.389
AST, U/L (median, IQR) 0–48 weeks	10.00 (1.50–29.00)	9.00 (2.00–28.50)	0.697
TBIL, µmol/L (median, IQR) 0–48 weeks	0.83 (-3.20–6.10)	-0.50 (-3.05–4.30)	0.497

HBeAg: Hepatitis B e antigen; HBV-DNA: Hepatitis B virus DNA; ALT: alanine transaminase; AST: aspartate aminotransferase; TBIL: total bilirubin; 0–48, weeks: the value detected at 0 weeks minus the value detected at 48 weeks.

### Fuzheng Huayu Recipe could reduce the production of serum HBeAg in CCl4-induced liver fibrosis HBV-Tg mice and induce HBeAg seroconversion

**FIGURE 2:**
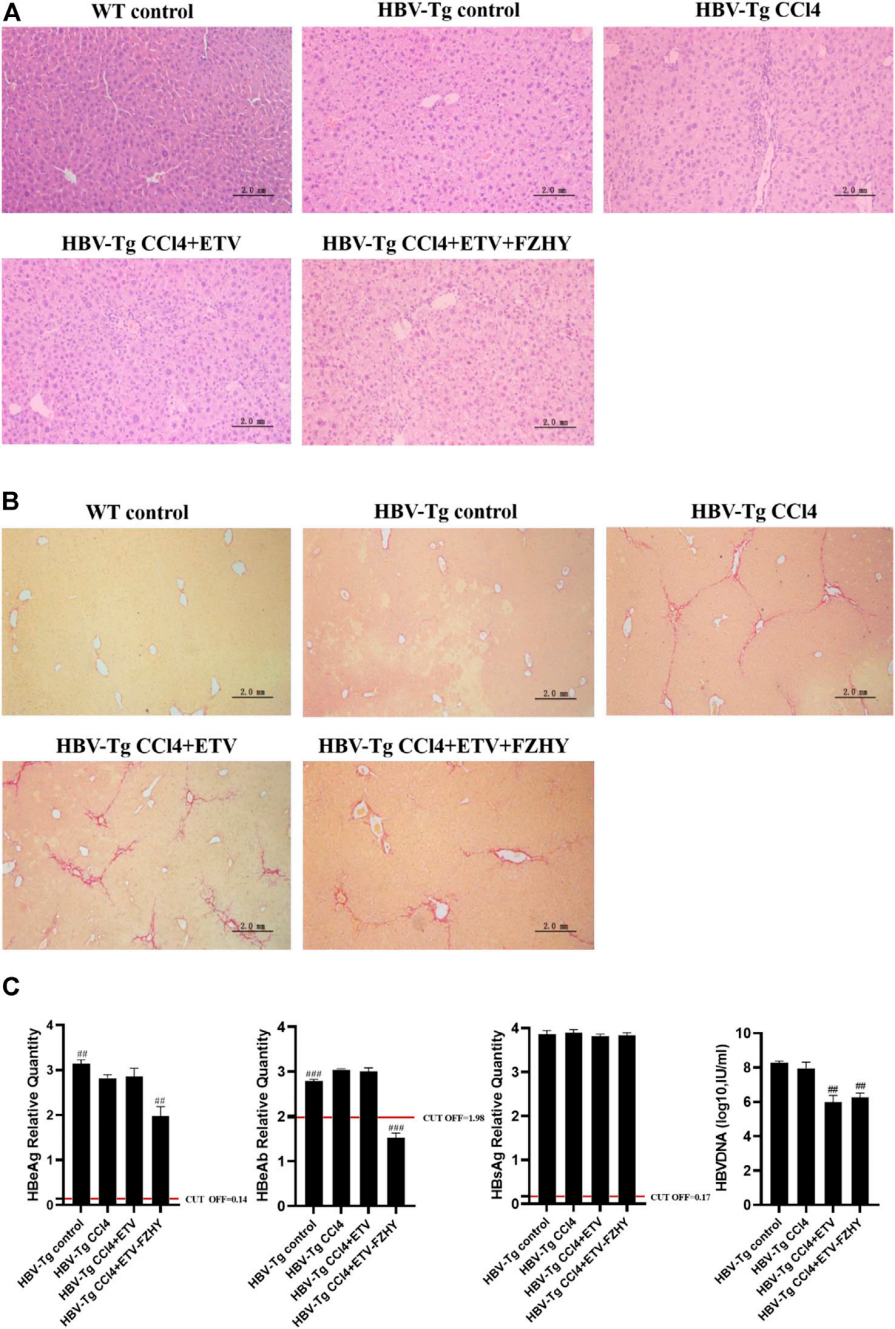
The pharmacological effect of FZHY on the expression of HBeAg, HBeAb, HBsAg, and HBV-DNA in CCl4-induced liver fibrosis HBV-Tg mice. **(A)**. HE staining. **(B)**. Sirius red staining. **(C)**. HBeAg, HBeAb, HBsAg, and HBV-DNA detection in HBV-Tg mice serum. Each group vs. CCl4-induced liver fibrosis group (HBV-Tg CCl4 group), ##*p* < 0.01, ###*p* < 0.001. Note: HBeAb cut-off value (COV) = 1.977, the result is positive if the sample value is less than COV value, and the result is negative if the sample value is greater than COV value.

To verify the pharmacological inhibitory effect of FZHY on HBeAg production, we induced CCl4 liver fibrosis HBV-Tg mice model and treated it with ETV or FZHY+ETV. The HE staining results showed that ETV and FZHY+ETV both could reduce hepatic lobular structure destruction, and significantly reduced pathological changes of hepatocytes (Figure 2A). The Sirius red staining results showed that ETV could reduce the extension of the fibrous septum in the portal area of liver tissue, and FZHY+ETV could regress collagen fiber more obviously: the fiber septum in the portal area of liver tissue was alleviated, and some of the fibers became thinner and narrower, or even disappeared (Figure 2B). These results suggested that FZHY could improve the inflammation damage and fibrosis degree of liver tissue in CCl4-induced liver fibrosis HBV-Tg mice.

Further serum detection results showed that, compared with CCl4-induced liver fibrosis HBV-Tg mice (HBV-Tg CCl4 group), HBeAg production and HBeAg seroconversion had no difference in ETV-administration mice (HBV-Tg CCl4+ETV group), but HBeAg production was significantly decreased and HBeAg seroconversion occurred in FZHY+ETV-administration mice (HBV-Tg CCl4+ETV+FZHY group). ETV-administration and FZHY+ETV-administration both had no effect on the production of HBsAg in CCl4-induced liver fibrosis group HBV-Tg mice. ETV-administration and FZHY+ETV-administration could both significantly decrease the production of HBV-DNA in CCl4-induced liver fibrosis group HBV-Tg mice, but there was no difference between ETV-administration and FZHY+ETV-administration. These results showed that FZHY could markedly decrease HBeAg production and promote HBeAg seroconversion, but had no effect on HBsAg and HBV-DNA production (Figure 2C).

### Fuzheng Huayu Recipe had no cytotoxicity on HepG2.2.15 cells and FZHY could significantly inhibit the production of HBeAg in HepG2.2.15 cells

**FIGURE 3:**
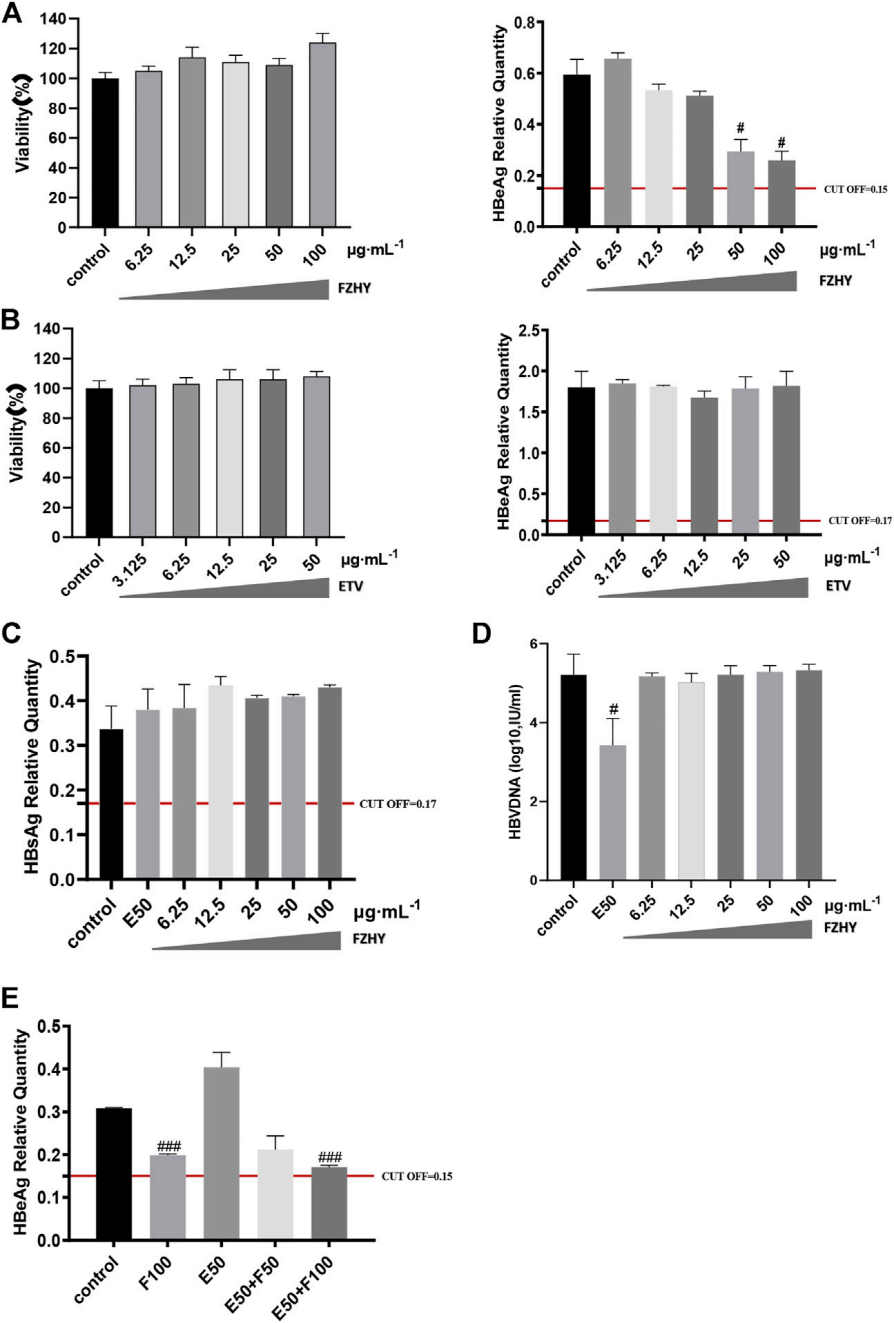
Cytotoxicity of FZHY on HepG2.2.15 cells and the effect of FZHY on the HBV expression in HepG2.2.15 cells. **(A)** Cytotoxicity and HBeAg production-inhibition effect of FZHY on HepG2.2.15 cells; **(B)** Cytotoxicity and HBeAg production-inhibition effect of ETV on HepG2.2.15 cells; **(C)** Effect of 50 μg/ml ETV (E50) and various concentrations of FZHY on HBsAg production; **(D)** Effects of 50 μg/ml ETV (E50) and various concentrations of FZHY on HBV-DNA production; **(E)** Effect of 50 μg/ml ETV (E50) combined with 50 or 100 μg/ml FZHY (F50 or F100) on HBeAg production. Each group vs. control group, #*p* < 0.05, ##*p* < 0.01, ###*p* < 0.001.

To further verify the pharmacological inhibitory effect of FZHY on HBeAg production, we used HepG2.2.15 cell line, which is constructed by transfecting the recipient cell HepG2 with the recombinant plasmid of HBV-DNA whole gene, and treated it with FZHY or ETV or FZHY+ETV. CCK8 was used to detect the cytotoxicity of different concentrations of FZHY (6.25, 12.5, 25, 50, 100 μg/ml) and ETV (3.125, 6.25, 12.5, 25, 50 μg/ml) on HepG2.2.15 cells. Compared with control group, both FZHY and ETV had no obvious cytotoxicity on HepG2.2.15 cells (Figures 3A,B). Compared with the control group, FZHY had a dose-dependent inhibitory effect on the production of HBeAg in HepG2.2.15 cells and 50 or 100 μg/ml FZHY showed the marked inhibition effect (Figure 3A), but different concentrations of ETV had no inhibitory effect on the production of HBeAg (Figure 3B). In order to better compare the pharmacological effects of ETV and FZHY, we chose 50 μg/ml as the subsequent experimental concentration of ETV. The further experiments results showed that, neither FZHY nor ETV had inhibitory effect on the production of HBsAg in HepG2.2.15 cells (Figure 3C). ETV had a significant inhibitory effect on the expression of HBV-DNA, but FZHY had no effect on the expression of HBV-DNA (Figure 3D). When 50 μg/ml ETV (E50) was combined with 50 or 100 μg/ml FZHY (F50 or F100), they both could significantly inhibit HBeAg production and E50 + F100 showed the better inhibition effect (Figure 3E). These results suggested that FZHY could specifically and markedly decrease the HBeAg production in HBV-infected hepatocytes. In the follow-up study on the mechanism of FZHY inhibiting HBeAg production, we selected 100ug/ml as the cell experimental concentration of FZHY.

### TOMM34 might be the key target of Fuzheng Huayu Recipe to inhibit HBeAg production

**FIGURE 4:**
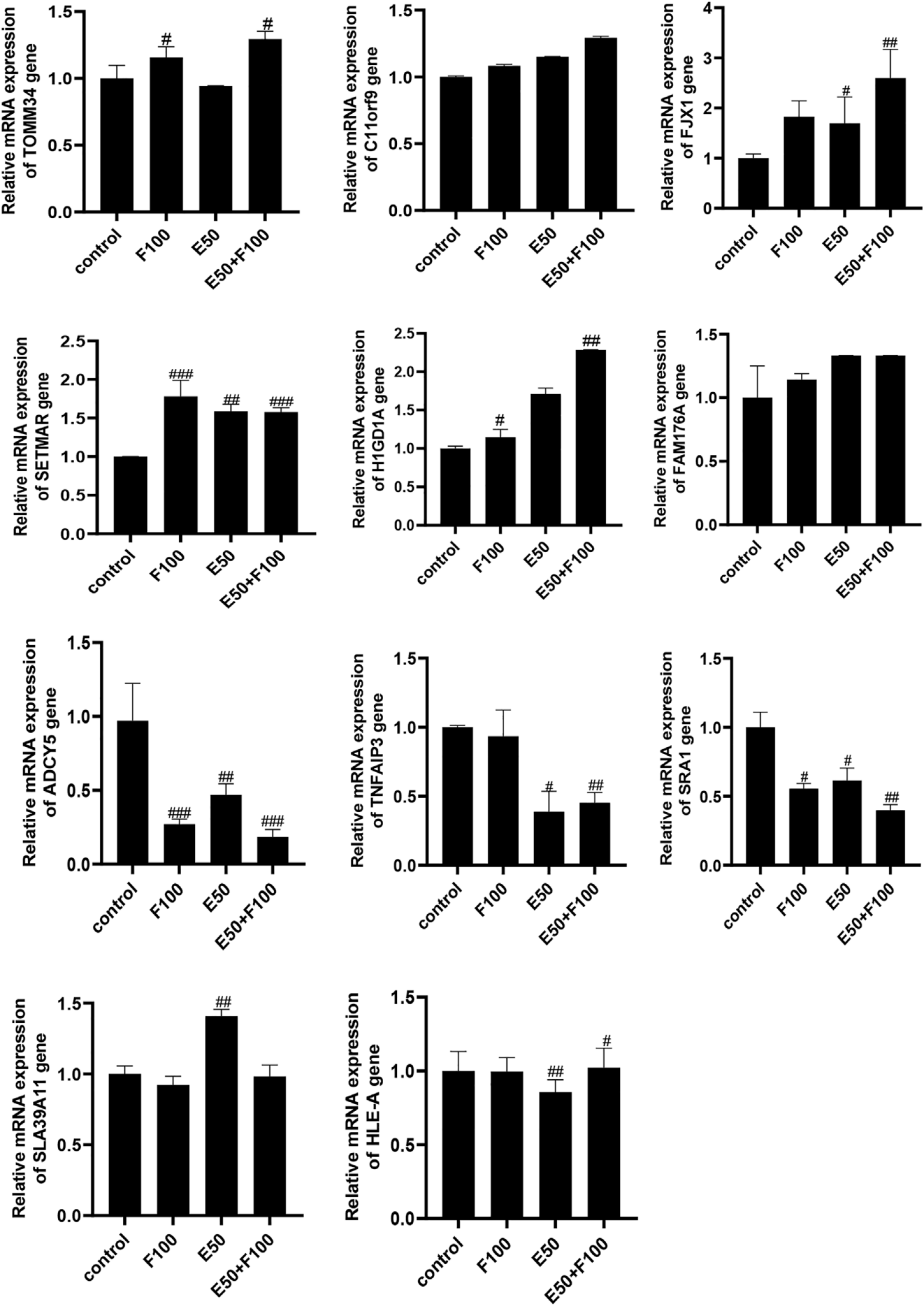
Screening of target genes for FZHY inhibiting HBeAg production. Each group vs. control group, #*p* < 0.05, ##*p* < 0.01, ###*p* < 0.001. F100: 100 μg/ml FZHY, E50: 50 μg/ml ETV, E50 + F100: 50 μg/ml ETV +100 μg/ml FZHY

Prof. Zhang Jiming’s team conducted a systematic study on the regulatory genes of HBV replication and HBeAg expression in 2012 (Liu, 2012). They discovered that there were 109 significantly differentially expressed genes in liver tissue of patients in inactive immune control period compared with patients in immune tolerance period, of which 54 genes were significantly up-regulated and 55 genes were significantly down regulated. In order to find out the target of FZHY inhibiting HBeAg production, we selected the genes related to HBeAg expression regulation, which were C11ORF9, FJX1, SETMAR, ADCY5, HIGD1A, FAM176A, TOMM34, SLA39A11, TNFAIP3, SRA1, and HLA-E, to screen the target of FZHY. The results showed that the TOMM34 gene relative expression levels in Hep2.2.15 cells had no difference between ETV group and control group, however, the expression of TOMM34 gene in FZHY or FZHY+ETV group were significantly higher than that in ETV group or control group (Figure 4). These results suggested that TOMM34 gene was related to the inhibitory effect of FHZY on the HBeAg production.

**FIGURE 5:**
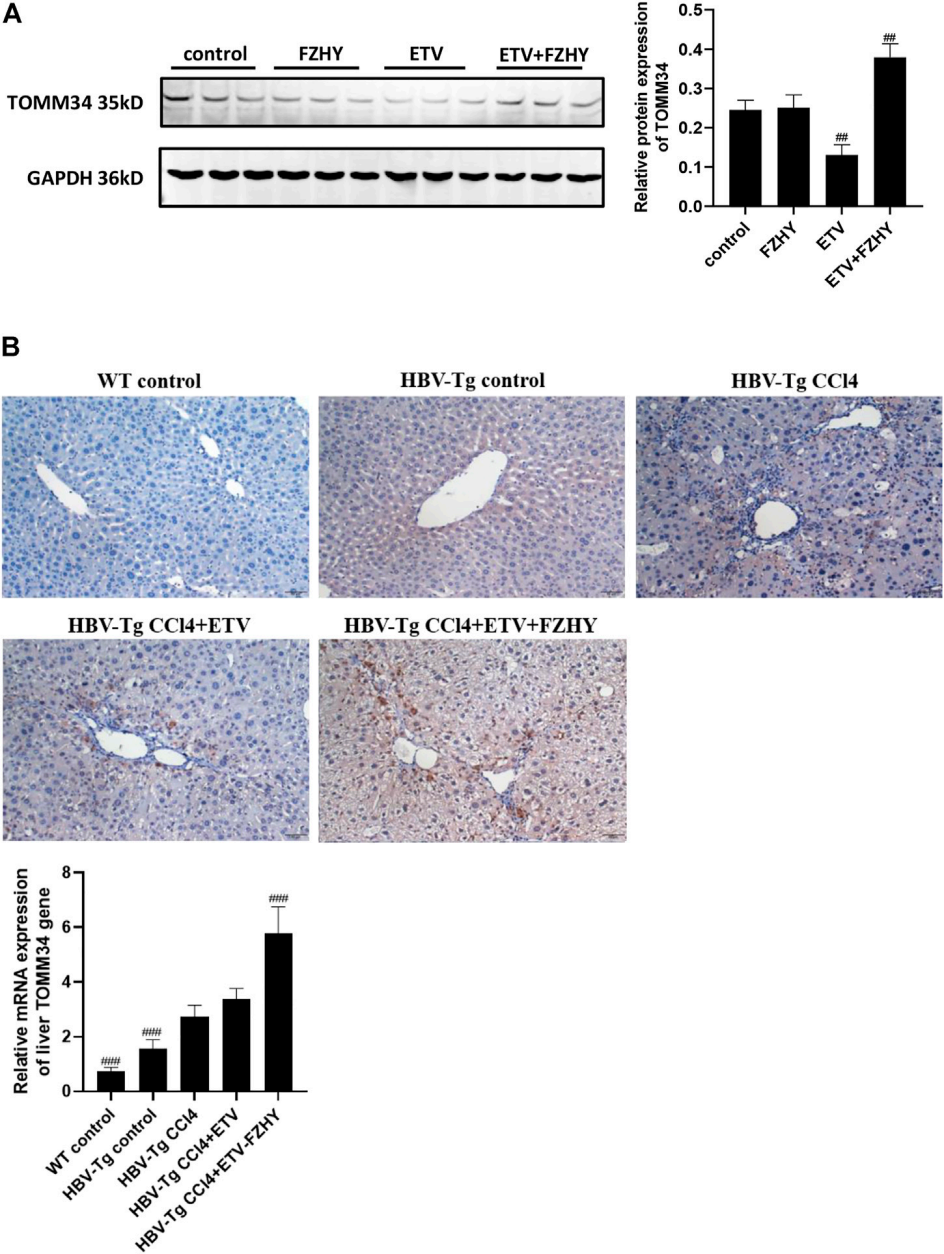
Effects of FZHY on the TOMM34 protein production in HepG2.2.15 cells and the TOMM34 gene expression in the liver tissues of CCl4-induced liver fibrosis HBV-Tg mice. **(A)** The TOMM34 protein production was detected by western blot assay. Each group vs. control group, ##*p* < 0.01. **(B)** The TOMM34 gene expression was detected by immunohistochemistry staining and qRT-PCR assay respectively. Each group vs. CCl4-induced liver fibrosis HBV-Tg mice group (HBV-Tg CCl4 group), ###*p* < 0.001.

We further detected the TOMM34 protein expression in Hep2.2.15 cells and HBV-Tg mice liver tissues. The western blot assay results showed that ETV decreased the TOMM34 protein expression in Hep2.2.15 cells, but FZHY+ETV significantly increased the TOMM34 protein expression (Figure 5A). The immunohistochemistry staining and qRT-PCR assay results also showed that ETV did not change TOMM34 protein and mRNA production in the liver tissues of CCl4-induced liver fibrosis HBV-Tg mice, but FZHY+ETV could markedly increase the production of TOMM34 protein and mRNA (Figure 5B). These results suggested that FZHY might inhibit HBeAg production by increasing TOMM34 gene expression.

**FIGURE 6:**
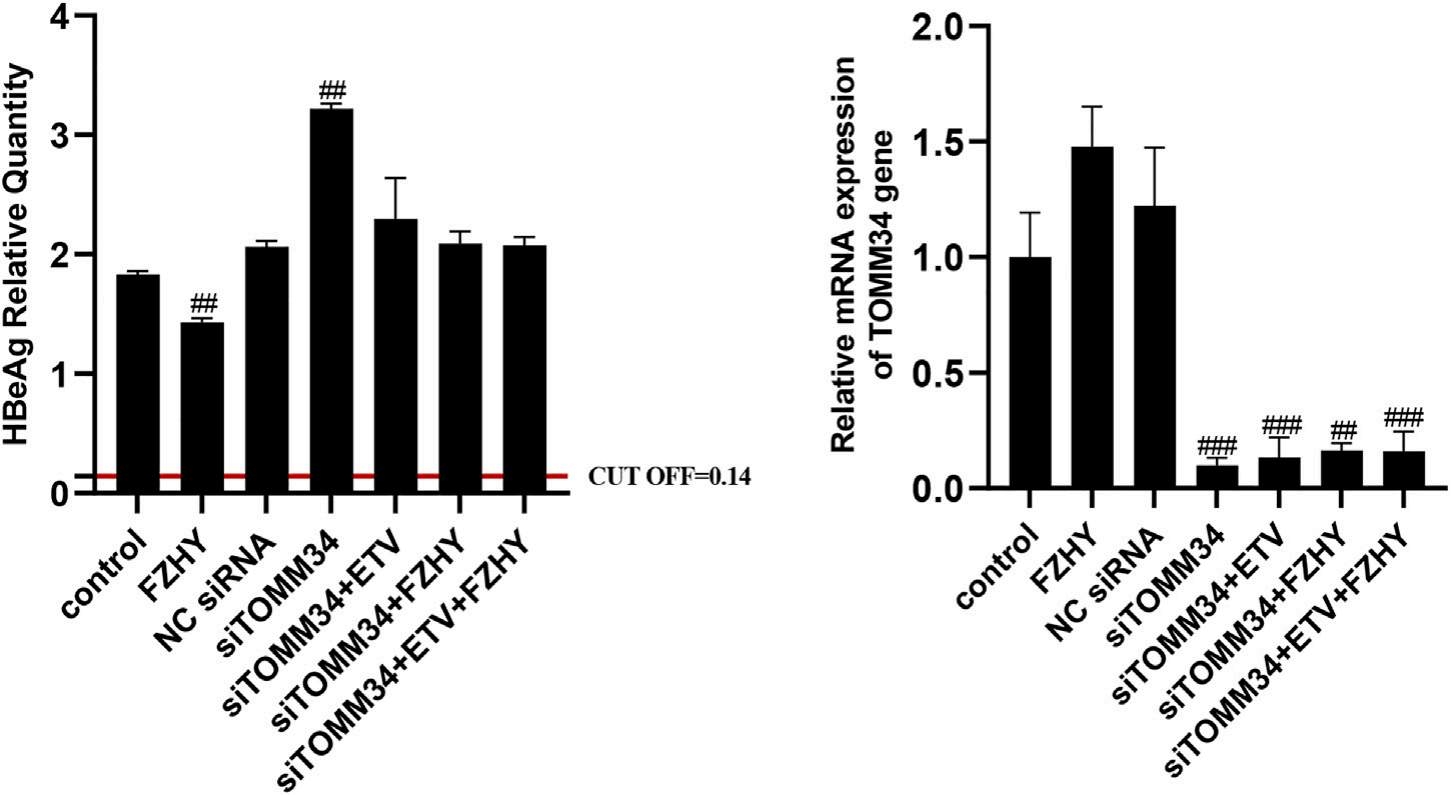
Effect of FZHY on HBeAg production in the TOMM34 gene-knocked down HepG2.2.15 cells. Each group vs. NC (negative control) siRNA group, #*p* < 0.05, ##*p* < 0.01, ###*p* < 0.001.

To further demonstrate whether FZHY inhibited HBeAg production by regulating TOMM34 gene expression, we knocked down the TOMM34 gene expression in HepG2.2.15 cells and administrate FZHY simultaneously. The results showed that the HBeAg production was obviously increased in HepG2.2.15 cells after TOMM34 gene expression was knocked down, and there was no differential production of HBeAg between between FZHY+ETV group and ETV group (Figure 6). These results suggested that TOMM34 was a key regulatory gene for FZHY to reduce HBeAg production in HBV-infected hepatocytes.

### The cytotoxicity of active compounds in Fuzheng Huayu Recipe on HepG2.2.15 cells and the active compounds’ effect on HBeAg production

**FIGURE 7:**
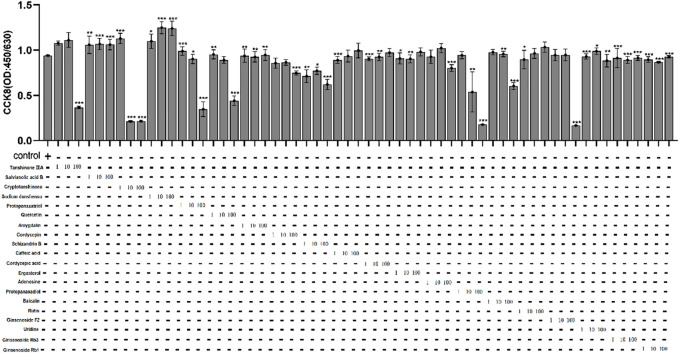
The cytotoxicity of 20 main active compounds of FZHY on HepG2.2.15 cells. Each group vs. control group, *p < 0.05, **p < 0.01, ***p < 0.001.

**FIGURE 8:**
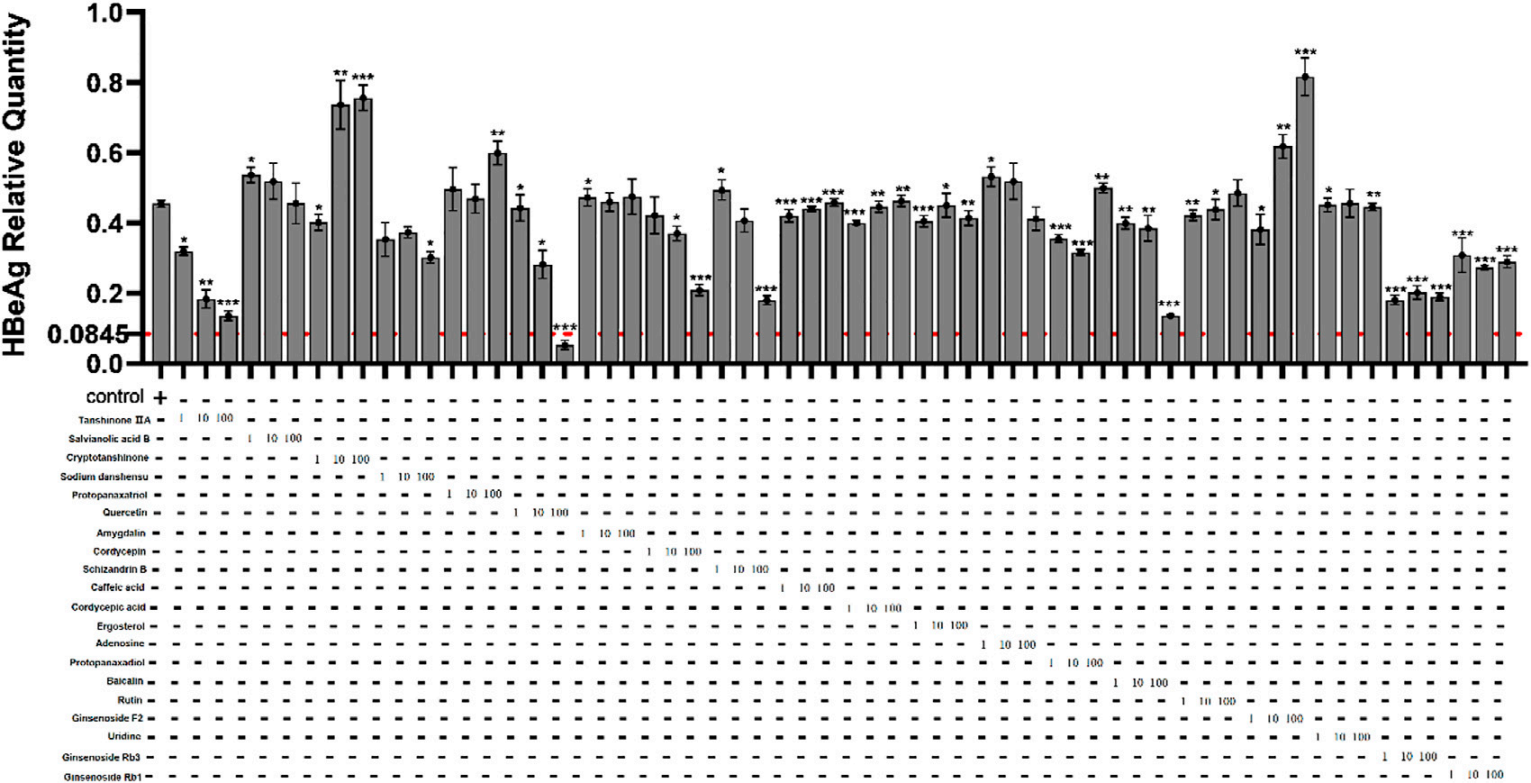
Effects of 20 main active compounds of FZHY on the HBeAg production in HepG2.2.15 cells. Each group vs. control group, *p < 0.05, **p < 0.01, ***p < 0.001.

In order to find out the main active compounds in FZHY that inhibit HBeAg production, the cytotoxicity of 20 active compounds in FZHY (caffeic acid, entomolic acid, ergosterol, adenosine, protopanaxadiol, baicalin, rutin, ginsenoside F2, uridine, tanshinone IIA, salvianolic acid B, cryptotanshinone, sodium tanshinol, protopanaxatriol, quercetin, amygdalin, cordycepin, ginsenoside Rb1, ginsenoside RB3, ginsenoside Rg3, and schisandrin B), was measured by CCK-8 assay (Figure 7) and the effect of these 20 active compounds in FZHY on the HBeAg production was detected by ELISA assay (Figure 8). The result showed that quercetin, cordycepin, baicalin, and schisandrin B could significantly inhibit HBeAg production at their low cytotoxic concentrations.

### Effects of quercetin, cordycepin, baicalin, and schisandrin b on HBeAg production and TOMM34 gene expression in HepG2.2.15 cells

**FIGURE 9:**
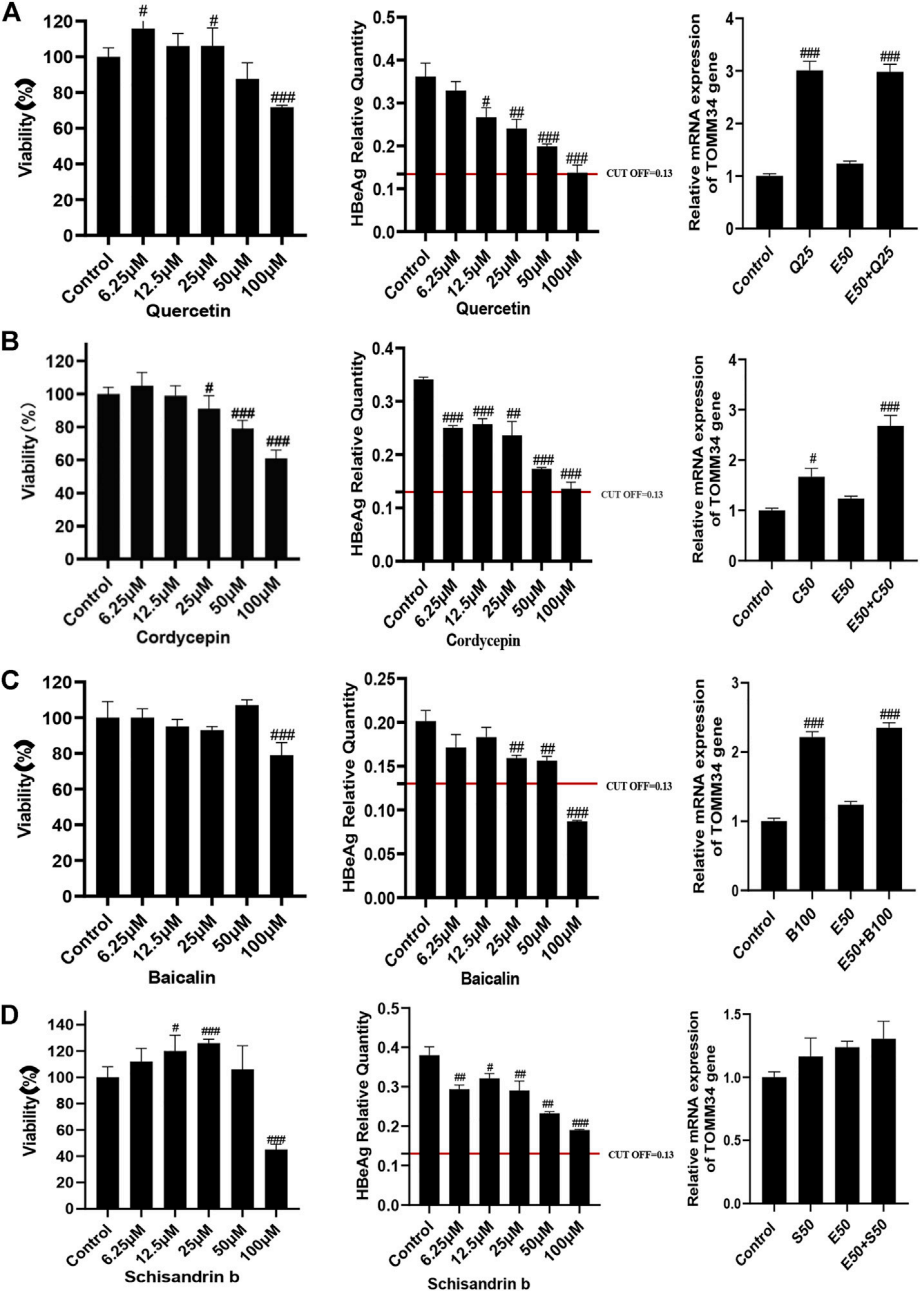
Effects of 4 active compounds of FZHY on the viability of HepG2.2.15 cells and the HBeAg production and TOMM34 gene expression in HepG2.2.15 cells. **(A)** Quercetin. E50: 50 μg/ml ETV, Q25: 25 μM Quercetin. **(B)** Cordycepin. E50: 50 μg/ml ETV, C50: 50 μM Cordycepin. **(C)** Baicalin. E50: 50 μg/ml ETV, B100: 100 μM Baicalin. **(D)** Schisandrin b. E50: 50 μg/ml ETV, S50: 50 μM Schisandrin b. Each group vs. control group, #*p* < 0.05, ##*p* < 0.01, ###*p* < 0.001.

We conducted further pharmacological screening experiments on quercetin, baicalin and cordycepin selected from the above experiment. The results showed that quercetin, cordycepin, baicalin, and schisandrin B could significantly inhibit HBeAg production in HepG2.2.15 cells in a dose-dependent manner. The results also showed that quercetin, cordycepin, and baicalin could significantly increase TOMM34 expression, but schisandrin B seemed had no obvious effect on TOMM34 gene expression. The results showed that quercetin, cordycepin, and baicalin could inhibit HBeAg production by increasing TOMM34 gene expression, and schisandrin B might inhibit HBeAg production by another regulatory pathway still unknown (Figure 9).

## Discussion

The degree of liver fibrosis in chronic hepatitis B affects the prognosis and management of patients. Antiviral treatment is related to the regression of liver fibrosis. Early HBeAg loss can reduce the risk of developing end-stage diseases, such as liver cirrhosis and liver cancer (Liaw et al., 2004). HBeAg may be used as an immune tolerance protein to help HBV virus escape the attack and clearance of the immune system. HBeAg clearance and HBeAg seroconversion after spontaneous or antiviral treatment show that HBV replication in patients is continuously controlled, patients’ immunity to HBV is improved, continuous response can be obtained, the proportion of liver decompensation is reduced and the survival rate is improved (Yu et al., 1997; McMahon, 2005). The relief of spontaneous persistent chronic hepatitis B can not only slow down the progress of liver fibrosis, but also reduce the degree of liver fibrosis (Hui et al., 2007). The mutation in the pre-core region (Kim et al., 1993) or the basic core promoter (BCP) (Kramvis and Kew, 1999) of hepatitis B virus can lead to the reduction of hepatitis B e antigen (HBeAg) expression, which makes HBeAg unable to be used as a therapeutic marker for the whole population of chronic hepatitis B. However, HBeAg loss and seroconversion are still considered as the satisfactory end point for chronic hepatitis B treatment (Chinese Society of Hepatology et al., 2015).

According to current clinical guidelines, the goal of chronic hepatitis B treatment is to improve the quality of life and survival rate by preventing the disease from developing into cirrhosis, decompensated cirrhosis, end-stage liver disease, liver cancer and death. Pegylated interferon and nucleoside/nucleotide analogues are currently approved first-line treatments for chronic hepatitis B virus infection and have been used for many years (EASL, 2012). However, these therapies can only inhibit HBV replication and are difficult to cure most chronic hepatitis B patients. The treatment of chronic hepatitis B patients with antiviral drugs such as nucleoside/nucleotide analogues not only has a high recurrence rate after drug withdrawal, but also may promote the development of virus resistance, so as to accelerating the deterioration of the disease. Entecavir (ETV) is a guanine nucleotide analogue. Although it has good safety, some studies have reported that it might cause lactic acid poison (Lange et al., 2009). The efficacy of immunomodulators such as conventional or pegylated interferon is very limited, and some side effects may occur during the treatment, such as flu like symptoms (Yang and Bertoletti, 2016). Therefore, we still need to find a safer and more effective method to treat chronic hepatitis B.

Clinically, the treatment of integrated traditional Chinese and Western medicine has achieved good therapeutic results. Fuzheng Huayu Recipe is one of the first-line traditional Chinese medicine prescriptions for the treatment of chronic hepatitis B liver fibrosis. Combined with the current first-line drug ETV, FZHY can significantly improve the clinical efficacy. Previous studies have shown that FZHY could play an anti-hepatic fibrosis role by inhibiting the activation of hepatic stellate cells (Luo et al., 2013), regulating the phenotypic polarization of macrophages (Zhang et al., 2020), and so on. The combination of Fuzheng Huayu Recipe and ursodeoxycholic acid could play a synergistic role on the basis of antiviral therapy. It could better improve liver function, inhibit inflammatory reaction and prevent the process of liver fibrosis in patients with hepatitis B cirrhosis (Li et al., 2018). Fuzheng Huayu Recipe combined with ETV could significantly improve liver function and liver fibrosis in patients with chronic hepatitis B (Wang et al., 2018). ETV combined with Fuzheng Huayu Recipe was effective in the treatment of patients with HBeAg positive decompensated liver cirrhosis. It could significantly improve liver function and effectively inhibit HBV replication (Zhan et al., 2018).

In 2012, Dr. Liu Jihong screened 83 patients with chronic hepatitis B in the study of “Screening and action mechanism of HBV replication regulatory genes in hepatocytes of patients with chronic hepatitis B″, including 22 patients in immune tolerance stage, 25 patients in immune clearance stage (positive chronic hepatitis B), 25 patients in immune activation stage (negative chronic hepatitis B) and 11 patients in inactive immune control stage. In addition, 6 healthy examinees were selected. The authors mainly observed the difference of gene expression in liver tissue under different chronic infection states in immune tolerance stage and immune control stage. Compared with patients in immune tolerance stage, there was significant difference in the expression of 109 genes in liver tissue of patients in inactive immune control stage (the expression of 54 genes was significantly up-regulated and 55 genes were significantly down regulated) (Liu, 2012). We conducted a pharmacological screening experiment of FZHY on the genes related to the regulation of HBeAg production, the results showed that translocase of the outer mitochondrial membrane 34 (TOMM34) was the main target of FZHY inhibiting HBeAg production.

TOMM34 is a subunit of mitochondrial transporter, which can transfer mitochondrial proteins from cytoplasm to mitochondria (Nuttall et al., 1997). The imbalance of TOMM34 expression has been discovered to be associated with the growth of many cancers, such as rectal cancer (Shimokawa et al., 2006), breast cancer (Aleskandarany et al., 2012), lung cancer (Gimenez-Xavier et al., 2017), hepatic cell carcinoma (Toraih et al., 2019; Zhang et al., 2021). However, the relationship between TOMM34 and liver fibrosis and cirrhosis has not been reported. Silencing the expression of TOMM34 can up-regulate the production of HBsAg, HBeAg, and nucleocapsid (Liu, 2012). Our research showed that FZHY and its monomer compounds quercetin, cordycepin and baicalin could inhibit the expression of HBeAg through up-regulating TOMM34 expression. This might be an important reason that the curative effect of FZHY+ETV was better than that of ETV alone in the treatment of hepatitis B liver fibrosis. The molecular mechanism of TOMM34 reducing HBeAg production needs to be further studied. This will provide more convincing evidence for TOMM34 as a therapeutic target for hepatitis B liver fibrosis.

## Conclusion

FZHY and its active compounds quercetin, baicalin and cordycepin could inhibit HBeAg production by promoting the expression of TOMM34 gene in HBV infected hepatocytes. FZHY+ETV combination therapy must be a recommended therapy for patients with HBeAg positive chronic hepatitis B and hepatitis B patients with liver fibrosis and cirrhosis.

## Data Availability

The original contributions presented in the study are included in the article/supplementary materials, further inquiries can be directed to the corresponding author.
